# Deficit Irrigation Applied to Lemon Trees Grafted on Two Rootstocks and Irrigated with Desalinated Seawater

**DOI:** 10.3390/plants12122300

**Published:** 2023-06-13

**Authors:** Josefa M. Navarro, Vera Antolinos, Pablo Botía, Juan M. Robles

**Affiliations:** Equipo de Riego y Fisiología del Estrés, Instituto Murciano de Investigación y Desarrollo Agrario y Medioambiental, 30150 Murcia, Spain

**Keywords:** *Citrus macrophylla*, sour orange, lemon, boron, sodium, chloride

## Abstract

The use of desalinated seawater (DSW) for irrigation in semi-arid regions is taking hold. Citrus tolerance to ions that predominate in DSW and water stress depends on the rootstock. Deficit irrigation was applied to DSW-irrigated lemon trees and grafted on rootstocks with different tolerance (*Citrus macrophylla* (CM) and sour orange (SO)). Plants were irrigated with DSW or Control treatment (distilled water), and, 140 days later, irrigation treatments were started: full irrigation (FI) or DI (50% of the volume applied to FI). After 75 days, differences between CM and SO plants irrigated with DSW and under DI were found. The higher concentrations of Cl^−^ and Na^+^ in CM and B in SO were the main causes of shoot growth reduction. The osmotic adjustment of CM plants was made possible by the accumulation of Na^+^, Cl^−^, and proline, but SO failed to adjust osmotically. In CM and SO plants, photosynthesis reduction was due to lower chlorophyll levels, but also to stomatal factors (CM plants) or alterations of the photochemical machinery (SO plants). Finally, unlike CM, SO had a good antioxidant system. In the future, knowing the different responses of CM and SO under these stressful conditions could be useful in citrus-growing areas.

## 1. Introduction

Climate change (CC) poses a threat to the ability of the agricultural sector to produce adequate food for the growing population. By 2050, irrigated food production will expand by more than 50%, which will require a 10% increase in water used for agriculture [1]. However, CC generates considerable uncertainty about future water availability in many regions of the world, where increased water scarcity will pose a major challenge to climate adaptation [2]. Under Mediterranean conditions, and on average, irrigation water needs will increase by 7.4% [3], which will lead to greater pressure on water resources, aggravating the situation in arid and semi-arid areas that suffer from water stress already [4,5]. Nowadays, Southeastern Spain, where climate conditions are those typical of semi-arid land, suffers from a high structural deficit of water, which makes it the region with the highest water deficit in the EU [6]. Currently, these areas are characterised by an increasing frequency of drought events, and by the scarcity of water resources that can prevent farmers from supplying enough water to their crops, which could lead to situations of deficit irrigation, mainly in the summer and spring seasons, affecting plant productivity [7].

In this context, and with the increasing difficulty to sustain agricultural production in arid regions such as the Mediterranean and Southeastern Spain, the use of alternative water sources is essential for ensuring the sustainability of agriculture in these vulnerable regions. In order to redress the limitation in the availability of conventional water as a resource in these areas, desalinated seawater (DSW) has been proposed as an option to adapt agriculture to the impacts of CC [8]. However, DSW does not seem to be problem-free, especially when compared with other conventional water resources. Its composition differs markedly from those of conventional water sources used for irrigation in Southeastern Spain: DSW has low mineralisation (some essential nutrients have been partially removed), and, in addition, the predominant ions that remain in DSW are Na^+^ and Cl^−^, whereas B is also present in high concentrations [9,10]. The quality of DSW can vary depending on several factors. Due to the chemical characteristics of the permeate, after RO processes, a post-treatment is required to re-mineralise and achieve an ionic balance; furthermore, depending on the type and intensity of post-treatments, and also on RO technology, the quality of DSW is highly variable [9]. There are no global regulations that define the DSW quality for crop irrigation, and this is simply expected to conform to the national potable water regulations [9]. In the case of Spain, the produced DSW fulfils the threshold of 1.0 mg/L for drinking water, and the use of DSW for irrigation could pose an agronomic risk, as RO permeates may have a boron concentration above the phytotoxicity thresholds of certain crops, such as woody crops (0.5 to 1.0 mg/L) [11].

On the other hand, in Southeastern Spain, where DSW is increasingly used for irrigation due to the scarcity of conventional waters, citrus species are amongst the most widespread crops, being essential for economic and social sustainability. It has been widely established that citrus trees are sensitive to the toxic effects derived from the accumulation of Na^+^, Cl^−^, and B in the leaves, which can damage plants by reducing the net assimilation of CO_2_, or the uptake of some nutrients [12,13,14,15]. The effects of high Na^+^ and Cl^−^ concentrations on citrus plants can be detrimental and have a significant impact on their growth, development, and overall productivity. These adverse effects on plant physiology are associated with low osmotic potential, specific ionic toxicity, and nutritional imbalances [16]: (1) Osmotic effect: The high presence of salts in the soil solution creates an osmotic imbalance, increasing soil osmotic potential and limiting water availability to the plant; to make an osmotic adjustment in order to avoid water stress, citrus plants use Na^+^ and Cl^−^ (accumulated mainly in the vacuole) as well as organic compatible osmolytes, such as proline, glycine betaine, sugars, etc. [17]. (2) Ion toxicity: The excessive accumulation of these ions in the plant can be toxic, since they may build up in the cytoplasm and in the chloroplast, therefore inhibiting enzymes involved in carbohydrate metabolism and photosynthetic process [18]. (3) Nutrient imbalances: Salinity affects the availability and uptake of essential nutrients by citrus plants since Na^+^ and Cl^−^ can interfere with the uptake of nutrients such as K^+^, Ca^2+^, and Mg^2+^, disrupting nutrient balance within the plant and leading to nutritional deficiencies [15]. Salinity can negatively impact the photosynthetic process in citrus plants by impairing chlorophyll synthesis, decreasing stomatal conductance, and inhibiting CO_2_ uptake. These factors reduce the ability of the plant to produce energy through photosynthesis, resulting in a decrease in growth and yield [14]. A secondary effect of salt stress in citrus plants is the triggering of oxidative stress [19], which can lead to the generation of reactive oxygen species (ROS), that can cause oxidative damage to cell membranes, proteins, and DNA, leading to cellular dysfunction and cell death [20].

Moreover, citrus trees are also sensitive to the drought stress that occurs in these areas. Water stresses, such as salinity, reduce the soil water potential and the ability of citrus plants to take up water, reducing and quickly trimming the growth of the plant [21]. Plants respond to water stress by trying to minimise the water loss through mechanisms such as the stomatal closure regulation, leaf rolling flexibility, and increase in the root/shoot ratio by creating a deeper and thicker root system, reducing leaf biomass, increasing cuticular resistance, and regulating root water conductivity [22,23]. Cell dehydration avoidance mechanisms are associated with osmotic adjustment and with cell wall hardening responses, contributing to the reduction of the water potential, while maintaining the cell’s turgor [24]. Cell dehydration tolerance mechanisms are characterised by the accumulation of osmoprotectants, antioxidants, and reactive oxygen species (ROS) scavengers [25]. Under water stress, the rate of photosynthesis rate is reduced by stomatal closure, membrane damage, and disturbed activity of various enzymes [26]. All these physiological and metabolic disturbances reduce plant growth and cellular metabolic processes of citrus plants, and therefore, the crop yield and fruit quality [27]. 

The tolerance of citrus to these environmental stresses (drought and high concentrations of Cl^−^, Na^+^, and B), is rootstock-dependent; hence, the use of the most tolerant rootstocks could be one of the agronomic strategies to take into account under stress conditions [16]. Common commercial citrus rootstocks have been classified based on their ability to restrict the uptake and/or the transport of Cl^−^, Na^+^, or B from the roots to the leaves, limiting its accumulation in the latter and minimising its toxic effect [12,28,29,30]. Additionally, differences in root distribution and growth, carbohydrates partitioning, water and nutrient uptake efficiency, as well as root hydraulic conductivity of the citrus rootstocks, have a marked influence on their tolerance to water stress [27,31,32,33].

Numerous studies have been carried out to evaluate the effect of water deficit on citrus using different rootstocks, although most of them have been made using good-quality waters [27,34,35]. However, under a scenery of water scarcity due to CC, more knowledge about the effects of deficit irrigation when alternative sources of water are used for irrigation is necessary. In recent years, the study of the effect of deficit irrigation using reclaimed waters is gaining importance [36,37], although, to date, the effects of deficit irrigation in citrus trees when DSW is used for irrigation are not known. The aim of this research was to study the effects of the deficit irrigation of citrus (lemon) plants irrigated with DSW and grown at high temperatures. In order to know which genetic material can be more suitable under a scenery of climate change, using DSW for irrigation and with periods of deficit irrigation, we studied the rootstocks that are most commonly used in lemon orchards in Southeastern Spain, namely *Citrus macrophylla* and sour orange (*Citrus aurantium*), which show different levels of tolerance to water deficit and the accumulation of Cl^−^, Na^+^, and B. These differences could modify the behaviour of SO- and CM-grafted plants (involving nutritional, physiological, and biochemical alterations) under deficit irrigation and when they are irrigated with desalinated seawater.

## 2. Results

### 2.1. Soil and Plant Water Status

**Figure 1:**
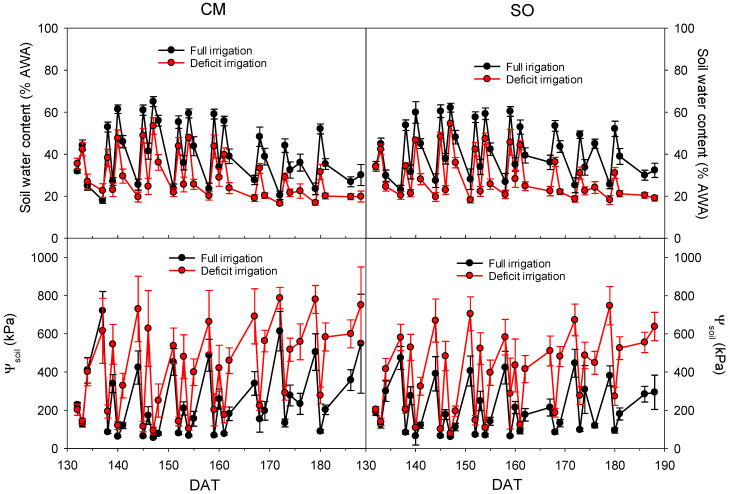
Evolution of the soil water content expressed as a percentage of AWA, and soil water potential (Ψ_soil_) after the irrigation treatments started in “Verna” lemon plants grafted on *Citrus macrophylla* (CM) or sour orange (SO) rootstocks. Irrigation treatments comprised full irrigation (FI) and deficit irrigation (DI, 50% of the volume applied to the FI treatment).

After 140 days of irrigating with an amount of water enough to allow for the drainage of the pots, deficit irrigation treatment (DI) started, and DI-irrigated plants were irrigated for 75 days more with 50% of the volume applied to plants under full irrigation (FI). The volumetric soil water content was measured by the soil moisture sensor throughout the experiment just before each irrigation event and the day after it. Soil water content expressed as the percentage of available water amount (AWA) is shown in Figure 1. The deficit irrigation treatments that were applied produced a decrease in the percentage of AWA (average of 30%) with regards to FI treatments (average of 41%), and these values ranged between 21–42% and 30–56% for DI and FI, respectively (Figure 1). The average soil water potential values (Ψ_soil_) were 249 and 134 kPa for DI and FI, respectively (values ranged between 133–477 kPa and 73–25 kPa for DI and FI, respectively). No differences between pots of CM or SO plants were found in the soil water content or Ψ_soil_ (Figure 1).

The lower amount of water due to the DI treatment produced a concentration of salts in the substrate (Table 1). Desalinated seawater had a higher EC than Control water, mainly due to higher concentrations of Na^+^, Cl^−^, and B (Table 2). Therefore, the irrigation with DSW for seven months significantly increased the EC of the substrates of the pots at the end of the experiment, mainly due to the accumulation of phytotoxic ions Na^+^, Cl^−^, and B (Table 1). Moreover, when DI was applied, the substrate of pots irrigated with DSW increased its EC due to the higher concentrations of Na^+^, Cl^−^, and B, but also of other nutrients, such as K^+^, Ca^2+^, Mg^2+^, NO_3_^−^, and SO_4_^2−^ (Table 1). In pots with both CM and SO plants, DI did not increase the concentrations of Na^+^ or Cl^−^ when plants were irrigated with the Control solution; nevertheless, they significantly increased when DSW was used for irrigation.

**Figure 2:**
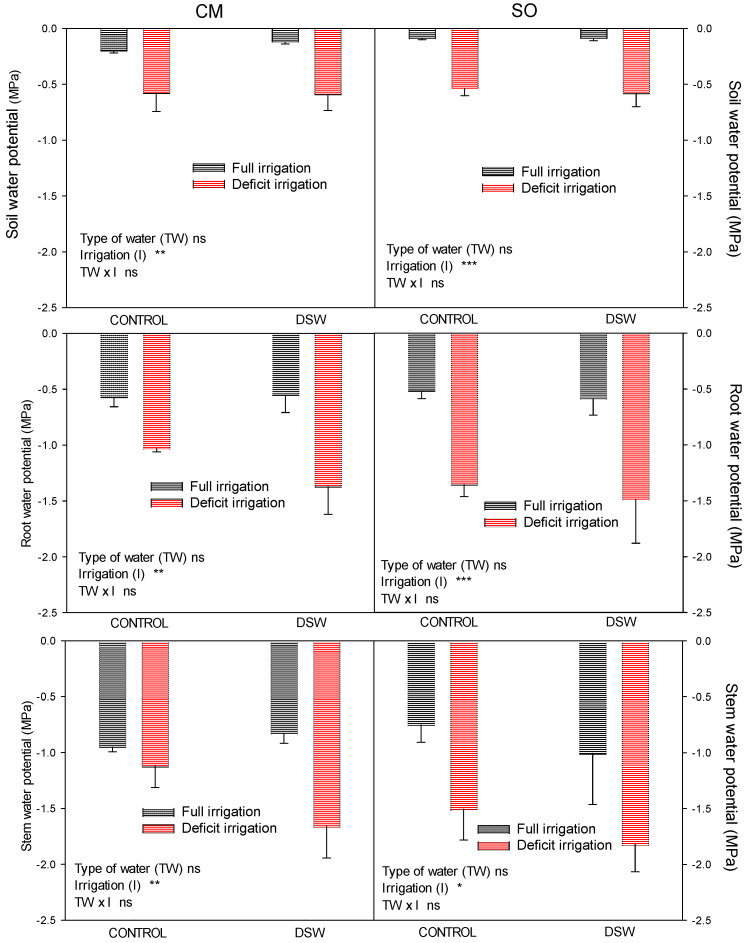
Water potential of soil, root, and stem measured at the end of the experiment in “Verna” lemon plants grafted on *Citrus macrophylla* (CM) or sour orange (SO) rootstocks. Plants were irrigated with two types of water (Control or DSW) and under two irrigation regimens: FI (full irrigation) and DI (deficit irrigation, 50% of the volume applied to the FI treatment). * *p* < 0.05; ** *p* < 0.01; *** *p* < 0.001; ns: not significant.

**Figure 3:**
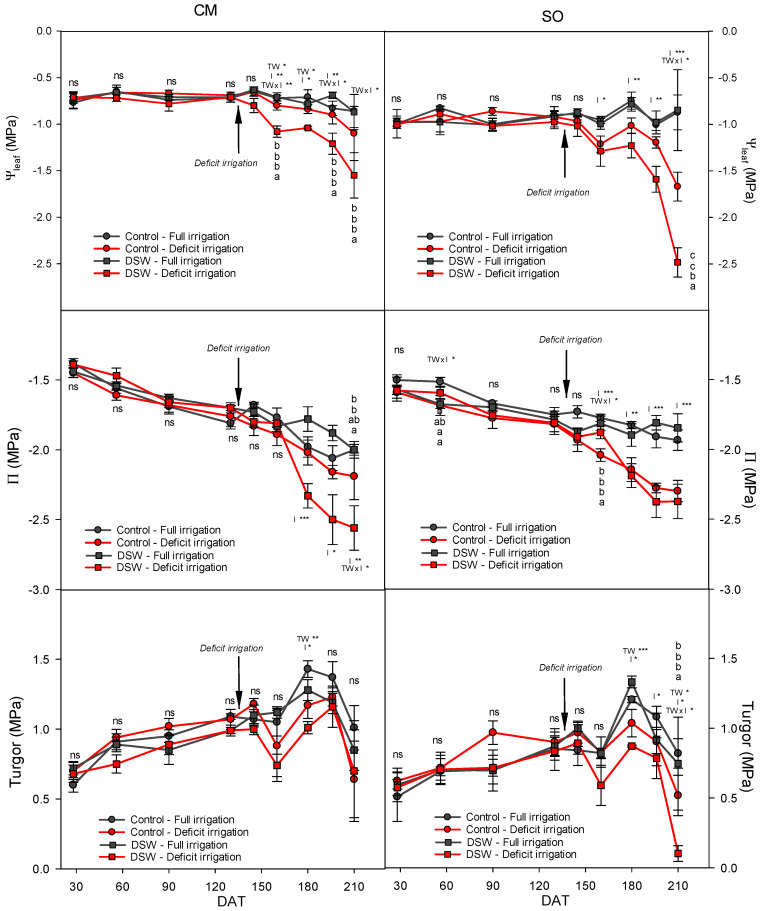
Leaf water potential (Ψ_leaf_), osmotic potential (Π), and leaf turgor measured throughout the experiment in “Verna” lemon plants grafted on *Citrus macrophylla* (CM) or sour orange (SO) rootstocks. Plants were irrigated with two types of water (Control or DSW) under two irrigation regimens: FI (full irrigation) and DI (deficit irrigation, 50% of the volume applied to the FI treatment). The arrow indicates the moment at which the deficit irrigation treatment began. * *p* < 0.05; ** *p* < 0.01; *** *p* < 0.001; ns: not significant. In each date, different letters indicate significant differences according to Duncan’s multiple range test at the 95% confidence level.

At the end of the experiment, water potential was measured in soil and plant (root, stem, and leaves). Similar soil water potential values were found in the pots of CM or SO plants irrigated with Control or DSW, with values ranging from −0.1 to −0.6 MPa (Figure 2). However, DI significantly decreased soil water potential in pots with both CM and SO plants, even though this decrease was higher in SO plants. The reduction of soil water potential due to the DI treatment produced a water potential decrease in the plant, firstly in the roots, secondly in the stem (Figure 2), and finally in the leaf (Figure 3). On the other hand, no differences in roots or stem water potential were found due to the irrigation with Control or DSW (Figure 2).

After deficit irrigation was started (140 DAT) by reducing the irrigation volume by 50%, a progressive decrease in Ψ_leaf_ due to DI was observed in CM, but mainly in SO plants (Figure 3). Additionally, this progressive decrease in Ψ_leaf_ produced a progressive decrease in osmotic potential (Π) in plants under DI treatment, although the behaviour of CM and SO plants irrigated with DSW was different. Whereas in CM plants Π decreased due to DI as a consequence of the decrease in Ψ_leaf_, in SO plants under DI and irrigated with DSW, the decrease in the osmotic potential was not enough, and turgor values of these plants were significantly decreased.

**Figure 4:**
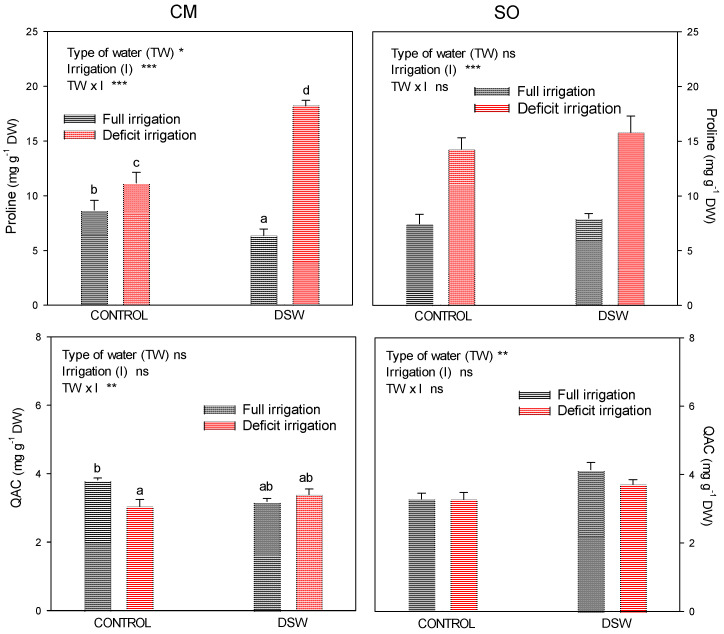
Proline and quaternary ammonium compounds (QAC) measured in leaves at the end of the experiment in “Verna” lemon plants grafted on *Citrus macrophylla* (CM) or sour orange (SO) rootstocks. Irrigation treatments comprised a FI (full irrigation) and DI (deficit irrigation, 50% of the volume applied to the FI treatment). * *p* < 0.05; ** *p* < 0.01; *** *p* < 0.001; ns: not significant. In each date, different letters indicate significant differences according to Duncan’s multiple range test at the 95% confidence level.

To study the contribution of organic solutes to the reduction of osmotic leaf potential, proline and quaternary ammonium compound concentrations were studied in leaves (Figure 4). In both rootstocks, CM and SO, proline concentrations were significantly increased when deficit irrigation occurred, but with some differences between them. Whereas in SO plants the increase in proline due to DI was similar in both Control and DSW treatments, in CM plants, the increase due to DI was significantly higher when plants were irrigated with DSW than in Control plants (Figure 4). Regarding QAC, no increase in these compounds was observed due to DI; however, SO plants irrigated with DSW accumulated more QAC than Control plants.

### 2.2. Plant Growth

As a consequence of the deficit irrigation treatment applied and also due to the irrigation with DSW, plant growth was modified in different ways. Seventy-five days after DI treatment was kicked off, CM and SO plants irrigated with DSW or under DI treatment showed a decrease in shoot growth (leaves and stems) (Table 3). The irrigation with DSW reduced both the stem and leaf growth due to the leaf size reduction. However, DSW did not decrease the leaf size of SO plants, and the reduction of plant growth with SO was only due to the effect of DSW on the stem (Table 3). On the other hand, DI treatment significantly affected the growth of CM and SO plants, reducing the leaf number and size in CM plants, but only affecting the number of leaves and stem weight in SO. The lower shoot growth due to DI treatment and the slight increase in root growth significantly increased the root/shoot ratio, whereas the total dry weights of CM and SO plants were not modified by DI.

On the other hand, although at the beginning of the experiment, plants had a uniform size on both rootstocks, after seven months, and regardless of the treatments, “Verna” lemon plants on SO rootstocks had shorter new growth stems (grown after the experiment was started), fewer leaves, and a lower leaf size, which produced a lower total leaf area and leaf weight than those found in plants grafted on CM rootstocks (Table 3).

At the end of the experiment, no differences in the percentage of leaves with visual injuries (necrosis along the tips and margins of leaves) were found between plants under full or deficit irrigation in both CM and SO plants. However, the irrigation with DSW significantly increased the percentage of foliar injuries in both CM and SO plants, with a slightly higher number of damaged leaves in SO that in CM plants, but with no significant differences.

### 2.3. Accumulation and Partitioning of Phytotoxic Elements

**Figure 5:**
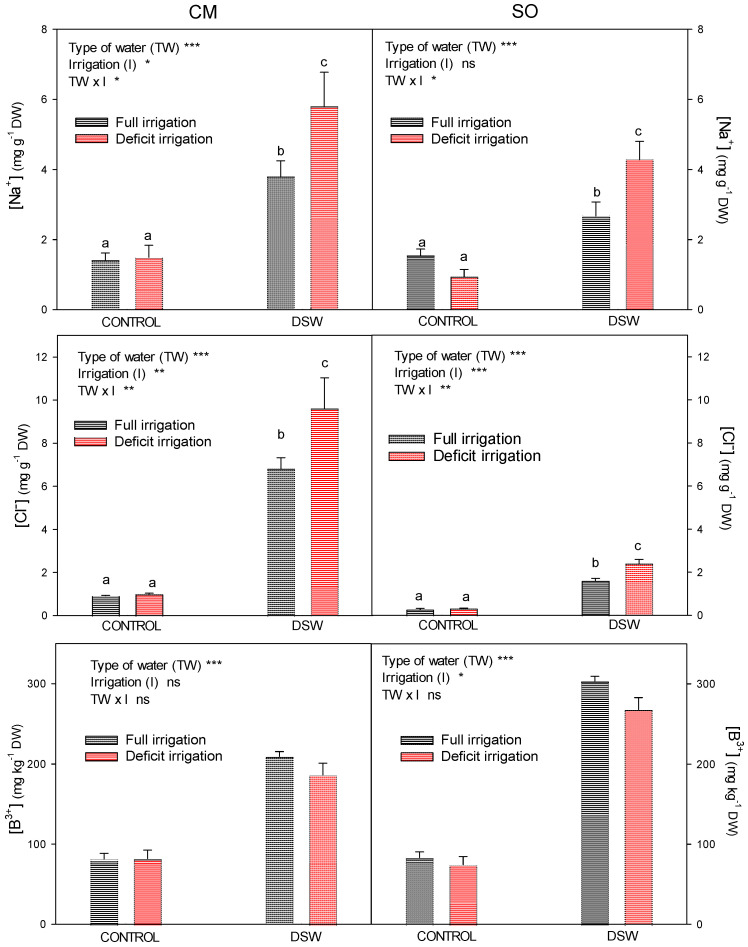
Effects of the irrigation with Control or DSW and under two irrigation regimens (full irrigation (FI) or deficit irrigation (DI)) on the Na^+^, Cl^−^, and B concentrations in leaves of “Verna” lemon plants grafted on *Citrus macrophylla* (CM) or sour orange (SO) rootstocks. * *p* < 0.05; ** *p* < 0.01; *** *p* < 0.001; ns: not significant. Different letters indicate significant differences according to Duncan’s multiple range test at the 95% confidence level.

“Verna” lemon plants grafted on CM or SO rootstocks were irrigated for seven months with DSW, with high concentrations of Cl^−^, Na^+^, and B in its composition (Table 2), which produced a strong accumulation of these phytotoxic elements in the soil (Table 1) and in the plant (Figure 5). After seven months, the Cl^−^, Na^+^, and B accumulation in the leaves of “Verna” lemon plants depended on the rootstock, the type of water used for irrigation, and the irrigation treatment (Figure 5).

In both CM and SO plants, the irrigation with DSW significantly increased the Cl^−^ and Na^+^ concentrations of the leaves, but the rootstock had a significant influence on its accumulation. Sour orange plants accumulated higher levels of Na^+^ than Cl^−^ in leaves, whereas leaves of CM plants had higher concentrations of Cl^−^ than Na^+^ (Figure 5). Deficit irrigation treatment also increased Cl^−^ and Na^+^, but only in plants irrigated with DSW. Regarding the accumulation of B, its accumulation in both CM and SO plants was mainly dependent on the type of water used for irrigation (Figure 5) since the use of DSW for plant irrigation for seven months produced an accumulation of B in leaves. Leaves of SO plants accumulated much more B than leaves of CM plants, whereas no significant effects on B accumulation were observed due to the DI treatment.

### 2.4. Physiological Responses and Oxidative Stress

Effects of the treatments on photosynthetic machinery (chlorophyll concentration, gas exchange, and chlorophyll fluorescence) were studied. In CM plants, photosynthetic rate (*A*) was significantly reduced by deficit irrigation, whereas in SO plants, *A* and transpiration (*E*) were reduced by DSW (Table 4). A decrease in stomatal conductance (*g_s_*) due to DI and the DSW was also observed in both rootstocks, although it was not significant. Leaf chlorophyll was reduced by DI in CM plants; however, in SO, the decrease in leaf chlorophyll due to DI was not significant, but it was reduced by DSW (Table 4). Deficit irrigation also reduced the efficiency of the antennas (F^’^_v_/F^’^_m_) in both CM and SO plants under DI, as well as the photochemical efficiency of PSII (Φ_PSII_) in CM plants (Table 4). Non-photochemical quenching (NPQ) significantly increased in both CM and SO plants under DI and irrigated with DSW.

**Figure 6:**
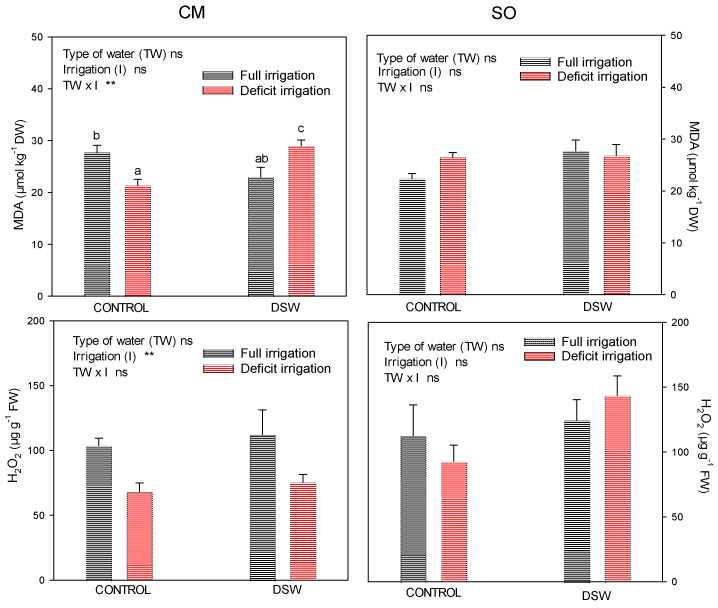
Malondialdehyde (MDA) and H_2_O_2_ measured in leaves at the end of the experiment in “Verna” lemon plants grafted on *Citrus macrophylla* (CM) or sour orange (SO) rootstocks. Irrigation treatments comprised a FI (full irrigation) and DI (deficit irrigation, 50% of the volume applied to the FI treatment). ** *p* < 0.01; ns: not significant. Different letters indicate significant differences according to Duncan’s multiple range test at the 95% confidence level.

In order to explore the oxidative damage generated by the irrigation with DSW and the DI regime that was applied, the production of H_2_O_2_ and a by-product such as malondialdehyde (MDA) was analysed (Figure 6). In addition, the antioxidant system was characterised by measuring the enzymatic activities of ascorbate peroxidase (APX), catalase (CAT), peroxidase, glutathione reductase (GR), and superoxide dismutase (SOD). The effect of the treatments on the production of MDA was different in both rootstocks, with no significant effect on SO plants but with an increase in MDA due to DI when DSW was used for irrigation in CM plants. With regard to the production of H_2_O_2_, no significant differences were found in SO plants due to the treatments, but a decrease in CM due to DI was observed.

Regarding the antioxidant enzyme system, no significant differences were found in the activities of the enzymes in CM plants (Table 5). However, the activities of antioxidant enzymes varied in SO plants with the use of DSW for irrigation and with the application of DI, with an increase in the activities of peroxidase and APX and a decrease in SOD when DSW was used for irrigation. When DI was applied in SO plants, the activity of APX and SOD was increased, whereas that of CAT and peroxidase was decreased (Table 5).

Generally speaking, a weak hormonal response was found in leaves of “Verna” lemon plants grafted on SO rootstock, and only a decrease in trans-zeatin was found in Control plants subject to DI with regard to FI (Table 6). In these plants, this cytokinin was also reduced when plants were irrigated with DSW under full irrigation. In plants grafted on CM rootstock, irrigation with DSW reduced trans-zeatin, but increased IAA with regard to Control plants. CM plants also decreased their GA4 concentration in leaves when they were subject to deficit irrigation (Table 6). No effects were found in ACC, IAA, ABA, JA, or SA due to the irrigation with DSW or to the deficit irrigation in both CM and SO plants.

## 3. Discussion

### 3.1. Osmotic Effects of Deficit Irrigation in Plants Previously Irrigated with DSW

Due to the scarcity of water resources in semi-arid areas, sometimes crops are subject to periods of water deficit. As a result of the increasingly frequent use of alternative sources of water, knowing the effects of deficit irrigation in citrus trees when DSW is used for irrigation is necessary. In this context, young citrus plants grafted on rootstocks with different salinity and drought tolerance were irrigated with waters of different qualities (Control and DSW) for 4.5 months. After that, a DI treatment was applied. When deficit irrigation was started, soil water content decreased, reducing the soil water potential with regard to FI treatments (Figure 1). At the end of the experiment, this effect was more significant in pots of SO plants than in those of CM plants (Figure 2). This lower soil water potential in pots of SO plants could be due to a higher transpiration rate in SO; however, these values were slightly higher in CM plants (Table 4). The lower soil water potential in SO could be due to the size of the plants, larger in SO than in CM (Table 3). Additionally, this higher soil water reduction in SO than in CM plants under deficit irrigation produced a higher reduction of plant water relations in these plants (Figure 3). At the end of the experiment, SO plants were more affected by DI than CM plants since Ψ_leaf_ values reached by them were more negative. This drop in Ψ_leaf_ produced a decrease in turgor in SO plants due to the reduction of Π, whose values were not enough to avoid such a decrease.

On the other hand, due to the lower amount of water in pots of DI treatments, the concentration of salts in these pots increased, which contributed to the drop of Ψ_leaf_ of these plants. The increase in salt accumulation in pots due to DI was higher when plants were irrigated with DSW, mainly due to the higher accumulation of Na^+^ and Cl^−^ in the substrates of these plants (Table 1). Previous results showed an increase in soil salinity after several months of irrigation with DSW, mainly due to the Na^+^, Cl^−^, and B accumulation in the soil [15].

As a consequence of the decrease in plant water potential, plants must lower their osmotic potential to maintain cell turgor and thus preserve cell metabolism. This decrease in Π is important in order to avoid the decrease in plant turgor; however, after DI was started, turgor values of DI plants were slightly under those of well-irrigated plants and, at the end of the experiment, the low Ψ_leaf_ values of SO plants under DI and irrigated with DSW produced a drop of turgor in these plants (Figure 3). However, CM plants under DI maintained their cellular turgor at values that were similar to those of well-irrigated plants, probably due to a higher osmotic regulation. In adult lemon trees grafted on CM, leaf turgor potential was maintained by osmotic and elastic mechanisms in plants under salinity conditions, but not under drought-stress conditions [38]. On the other hand, it is known that under drought conditions, net uptake of certain inorganic ions into plant cells could be enhanced on citrus seedlings to increase the presence of inorganic solutes that facilitate osmotic adjustment [7]. In our experiment, the reduction in Π observed in plants under DI was due to the accumulation of phytotoxic elements, such as Na^+^ and Cl^−^ (Figure 5), that contributed to the osmotic adjustment in both CM and SO plants, although the low Cl^−^ concentration in SO plants irrigated with DSW with regard to CM plants suggests a low contribution of Cl^−^ to osmotic adjustment in these plants. The synthesis of organic solutes, mainly proline and quaternary ammonium compounds (QAC), can also play an important role in the osmotic adjustment as a response of plants to stress [39,40]. In this experiment, plants under DI, but mainly those irrigated with DSW, used proline as an osmolyte to reduce the osmotic potential of their leaves (Figure 4). Although proline was increased under DI in both CM and SO plants, once again, in SO plants irrigated with DSW, the increase in proline was lower than in CM plants, so the better osmotic adjustment shown by CM plants with regard to SO plants irrigated with DSW and under DI was probably due to the higher Cl^−^ and proline concentrations. In spite of the results notified by other authors, which determined that QAC were the only organic solutes involved in the adaptation of citrus to B toxicity [41], we found no role of QAC in the osmotic adjustment process of plants irrigated with DSW.

### 3.2. Accumulation of Phytotoxic Elements in the Plant

The accumulation of phytotoxic elements (Na^+^, Cl^−^, and B) in the plant (Figure 5) due to the irrigation with DSW for 4.5 months before the DI was started could be decisive in their behaviour during the subsequent period of water deficit. In fact, among the causes of shoot growth reduction in DSW-irrigated plants were the toxic effects due to the high concentrations of Cl^−^, Na^+^, and B in the nutrient solution (Table 2). Concentrations of Cl^−^ and Na^+^ in DSW were above 152 and 115 mg L^−1^, respectively; these values were proposed as the thresholds to produce injury in citrus [42,43]. Boron concentrations in the DSW nutrient solution also exceeded the threshold of 0.50 mg L^−1^ proposed for citrus trees [10]. The irrigation with DSW throughout the experiment produced a strong accumulation of these phytotoxic elements in the soil (Table 1) and, consequently, in the plant (Figure 5), which could affect its growth in a negative way, since high concentrations of these elements on leaves of citrus plants reduce plant growth due to their sensitivity to B and salts [12,15,16,44].

However, the accumulation of Cl^−^, Na^+^, and B in citrus plants was dependent on the rootstock, as we have previously reported [15]. After seven months of irrigation with DSW, foliar Na^+^ concentrations on CM plants far exceeded the phytotoxicity threshold of 2.5 mg g^−1^ DW proposed by Grattan et al. [43], and the DI treatment applied during the last 75 days significantly increased these concentrations (Figure 5), since soil Na^+^ concentration in DI treatments doubled that found with FI (Table 1). Nonetheless, foliar Na^+^ concentrations on SO plants irrigated with DSW and full irrigation were within the limit of this phytotoxicity threshold, which was only surpassed when DI was applied.

The concentration of Cl^−^ in DSW (Table 2) was twice as high as the injury-producing threshold in citrus according to Grattan et al. [43], and, after using it for irrigation for seven months, Cl^−^ accumulated in soils irrigated with DSW was more than five times higher than that found in Control soils (Table 1). However, in spite of these high Cl^−^ concentrations in the irrigation water and in the soil, foliar Cl^−^ concentrations in DSW-irrigated CM plants with full irrigation were only slightly above the leaf toxic threshold of 6 mg g^−1^ DW proposed by Romero-Trigueros et al. [45], and only when these plants were subject to deficit irrigation Cl^−^ levels were close to the threshold of 10 mg g^−1^ DW proposed by Grattan et al. [43]. However, in SO plants irrigated with DSW, foliar Cl^−^ levels were far below the toxic threshold of 6 mg g^−1^ DW, even in plants under DI (Figure 5). On the other hand, concentrations were expressed on a leaf-DW basis; therefore, Cl^−^ and Na^+^ increase under DI could not have been due to a dehydration process in the leaves, but to the effect of the concentration of salts in the soil derived from the reduction of available soil water.

The high B concentration in DSW also produced a high accumulation in the soils irrigated with this type of water, with levels that were approximately five times above those found under Control treatments (Table 1). As a consequence, CM plants irrigated with DSW had a foliar concentration of B that ranged between 100 mg kg^−1^ DW (the threshold above which damage can occur) and 250–260 mg kg^−1^ DW (range in which toxicity occurs in citrus; [46]); however, plants grafted on SO had foliar B concentrations above this high threshold of toxicity (Figure 5). Each rootstock had a different response to the high B concentration of irrigation water, and this response was dependent on the B accumulated in the leaves, as has been seen in previous studies [15,29,47]. In spite of this, at the end of the experiment, B concentration in soils under DI increased by 30% with regard to full irrigation, and unlike what was observed with Na^+^ and Cl^−^, B concentrations were not increased in leaves by deficit irrigation (Figure 5).

According to all of these results, Cl^−^, Na^+^, and B concentrations were high enough to produce foliar injuries in DSW-irrigated plants, but when DI was applied, Cl^−^ and Na^+^ concentrations reached higher levels, which contributed to the slight decline in plant growth in these plants (Table 3). However, in SO plants irrigated with DSW, B accumulation was the main cause of the plant growth reduction observed in these plants regardless of the irrigation treatment. On the other hand, an interaction of drought and B toxicity has been established when plants are exposed to both stresses in combination [48], with elevated resistance to drought stress in B-nutrition-rich plants mediated by an improvement in sugar transport, photosynthetic efficiency, hormone synthesis, lipid metabolism, flower retention, pollen formation, seed and grain production, and seed germination [49]. This synergistic effect could help plants to deal with the stress imposed by DI and high B concentrations due to irrigation with DSW.

### 3.3. Physiological and Biochemical Responses of Rootstocks to Salt/Drought Stresses

The plant behaviour of CM and SO plants irrigated with DSW and under DI was affected by some different physiological, biochemical, and nutritional alterations. One of the most important affected processes that are directly related to plant development is the photosynthesis rate (*A*). In our experiment, *A* was reduced in SO plants by the irrigation with DSW due to a lower stomatal conductance, although in CM the photosynthetic rate reduction by DI was not attributed to stomatal factors (Table 4). Abscisic acid (ABA) is involved in the regulation of stomatal closure as an adaptive response under drought conditions [50]. However, no significant changes in ABA concentrations were found in CM or SO plants under DI treatments (Table 6). Hormonal profiling in citrus leaves revealed that ABA levels strongly increased in water-stressed plants, whereas heat stress repressed ABA accumulation, probably avoiding stomatal closure and keeping high transpiration rates to cool the surface of the leaves [51]. These authors pointed out that during a combination of drought and heat stress, other mechanisms must be involved in regulating stomatal responses since they found that stomatal conductance decreased despite the reduction of ABA levels under these situations. It has been suggested that H_2_O_2_ and jasmonic acid (JA) could signal stomatal closure in plants subject to combined drought and heat independently of ABA signalling [52]. However, no increases in JA nor in H_2_O_2_ have been found in this experiment in response to DI (Table 6).

In any case, in the *A* reduction in SO and CM plants due to DSW and DI, respectively, other non-stomatal factors could have intervened, as it has been previously established in citrus plants [14,15,53,54]. Some of these factors are the reduction of the chlorophyll concentration, alterations in the carboxylation efficiency, reduced activities of photosynthetic enzymes, alterations in the photochemical efficiency of photosystem II, an impaired electron transport capacity, and alterations to the leaf structure and chloroplast ultrastructure [54,55,56]. The *A* reductions observed on CM plants due to DI, and due to DSW on SO plants, were partly due to the reduction of chlorophyll concentration under these treatments (Table 4). However, some alterations in the photochemical machinery of the leaves were also implicated. The study of chlorophyll fluorescence can provide information on the possible alterations that can occur in the processes related to the photochemistry of PSII. It has been established that *A* reductions due to water stress led to an excess of irradiation energy and to a reduction in photochemical quenching (qP), reducing the maximum quantum efficiency and the efficiency of light-harvesting centres (F’_v_/F’_m_) [57]. The conversion efficiency of the light energy captured by the photosynthetic pigments into photochemical energy in PSII of the chloroplasts (Φ_PSII_) was decreased by DI in CM plants, and this reduction in Φ_PSII_ could be due to changes in qP, and/or in F’_v_/F’_m_ [58]. Our chlorophyll fluorescence results showed that F’_v_/F’_m_ was decreased by DI in both CM and SO plants. The reduction of Φ_PSII_ in CM due to DI was not due to changes produced in qP, which indicated that the loss of quantum efficiency by PSII was not due to damage in the electron transport chain, but to damage in the light-harvesting complex [58]. On the other hand, in both CM or SO plants, irrigation with DSW did not alter either F’_v_/F’_m_ or Φ_PSII_, but under the most stressful conditions (plants irrigated with DSW and under DI), both CM and SO plants increased their NPQ in order to protect the PSII reaction centres of harmful excess excitation energy due to the reduction of the energy utilised in the photosynthesis process (Table 4). The non-photochemical quenching (NPQ) is a protective mechanism that occurs when light energy absorption exceeds the capacity for light utilisation in photochemistry processes, and this excess excitation energy can be harmlessly dissipated as heat through molecular vibrations [59].

On the other hand, in both CM and SO plants irrigated with DSW, the A/Φ_PSII_ ratio was decreased (Table 4), which means that the excess of electrons that were not utilised in metabolic processes could be accepted by O_2_, generating reactive oxygen species (ROS) in the leaves [60]. To deactivate ROS, plants have to increase the activity of specific enzymes, but when this does not occur, the overproduction of these species results in oxidative damage, since ROS react with many different molecules yielding MDA, which is used as a marker for oxidative damage [61]. The decrease in the A/Φ_PSII_ ratio and the increase in MDA in the leaf of CM plants irrigated with DSW and under DI (Figure 6) suggests that these “Verna” lemon plants have an inefficient antioxidant system and were not able to cope with the produced ROS. However, in spite of the decrease in the A/Φ_PSII_ ratio of SO plants irrigated with DSW and under DI, no increase in MDA was observed in these plants, which suggests that SO has a better antioxidant system than CM rootstock. In other studies, SO exposed to a high B concentration showed an efficient antioxidant system able to deal with ROS [41]. When some of the specific enzymes used to deactivate the ROS were studied in this experiment, different responses were observed in CM and SO plants. Whereas no effect of the oxidative stress generated by DSW or by DI was found on CM plants, the responses of SO to oxidative stress were based on increments in the activities of APX and peroxidase when the stress was generated by DSW, or on the increase in the activities of APX and SOD when the stress was generated by deficit irrigation. On the other hand, the nutritional status of B may affect the sensitivity of plants to drought, because this element is involved in the detoxification of ROS, playing a protective role in preventing photooxidative damage catalysed by ROS in chloroplasts [49,62], and conferring protection against oxidative damage of membranes, controlling the overproduction of H_2_O_2_ and alleviating the negative consequences of electrolyte leakage in the plasma membrane [49]. In this sense, the higher B concentration in SO plants with regard to CM plants (Figure 5), and a higher response of the specific enzymes that are used to deactivate the ROS, could have contributed to a better antioxidant response of these plants under DI treatment. All of these data corroborate the different behaviour of CM and SO plants. In both of them, A/Φ_PSII_ decreased, generating an increase in ROS; nevertheless, only in SO plants ROS were efficiently deactivated by an efficient antioxidant system, and no MDA was produced in these plants.

These data verified that each rootstock has a different response to the stress generated by the irrigation with DSW or by DI and that this response was dependent on the phytotoxic elements accumulated in the leaves, as well as its specific toxicity.

## 4. Materials and Methods

### 4.1. Experimental Design

One-year-old “Verna” lemon (Citrus limon Burm. f. cv. Verna) trees grafted on two different rootstocks, *Citrus macrophylla* (CM) and sour orange (*Citrus aurantium*) (SO), were used in this experiment. Plants were grown in 3 litre capacity pots filled with a substrate composed of a mixture of silica filtration sand and clay loam soil (soil:sand 3:1, *v*/*v*). The experiment was carried out in a growth chamber under the same experimental conditions previously described in Navarro et al. [15]: 14/10 h day/night cycle RH of 55/85% and high temperature (35/27 °C) to simulate the extreme conditions of CC. After plants were acclimatised to these conditions for two weeks, they were irrigated with two types of irrigation water (supplemented with Hoagland nutrients [63]: distilled water (Control) and desalinated seawater (DSW) obtained from the desalination plant of Escombreras (Murcia, Spain). The final mineral concentrations in the two types of nutrient solutions are shown in Table 2. Plants were irrigated three times per week with a volume that was sufficient to produce leachate from the bottom of all of the pots.

After 140 days of irrigating with the two types of water, two different irrigation treatments were applied: full irrigation (FI, as described above) or deficit irrigation (DI, a 50% volume of nutrient solution applied to FI). The experiment was finished 75 days after DI treatments were initiated. The experiment was laid out in a completely randomised design and consisted of factorial combinations of two factors: type of water (Control and DSW) and irrigation (full irrigation (FI), or deficit irrigation (DI)), presenting a total of four treatments with four replicates per treatment. These four treatments were applied to “Verna” lemon plants grafted on CM and SO rootstocks, for a total of 32 pots.

### 4.2. Soil and Plant Water Status

After DI treatments were started, volumetric soil water content (θ_v_) was monitored every other day by inserting a theta probe (Model ML2X, Delta-T Devices) into the top of the pot. Dielectric soil moisture sensor readings were calibrated by comparing soil moisture of each pot with the gravimetric calculation. In order to estimate soil water potential (Ψ_soil_) from the volumetric soil water content values, a soil water retention curve of the substrate used in the experiment was elaborated (Ψ_soil_ [kPa] = 23,301 e^−24^ θ_v_, R = 0.90, *p* < 0.0001). At the end of the experiment, the soil water content of each pot of an individual plant was also determined using the gravimetric method. Soil water potential was measured with a dew point potential meter (WP4C, Decagon Devices, Pullman, WA, USA).

Leaf water potential (Ψ_leaf_) was periodically measured in mature fully expanded leaves with a Schölander-type pressure chamber (model 3000; Soil Moisture Equipment Corp., Santa Barbara, California, USA), following the recommendations of Turner [64]. After Ψ_leaf_ measurement, leaves were immediately frozen and stored at −20 °C to determine the leaf osmotic potential (Π) with a Wescor 5520 vapour pressure osmometer (Wescor, Logan, UT, USA). Leaf turgor potential was calculated as the difference between Ψ_leaf_ and Π.

### 4.3. Plant Growth

At the end of the experiment, roots were carefully separated from the substrate and washed with distilled water. Shoots were separated into leaves and stems, which were also divided into lateral (growth after transplanting) and old stems. The leaf area of each plant was measured using a leaf area meter (model LI-3100, Li-Cor, Lincoln, NE, USA). Each plant material fraction was weighed fresh and after being in the oven for 48 h to determine the dry weight (DW).

### 4.4. Plant Gas Exchange and Chlorophyll Fluorescence

Leaf gas exchange and chlorophyll fluorescence measurements were performed in parallel in the youngest fully expanded leaf of each plant, as previously described by Navarro et al. [15]. Leaf gas exchange was measured using a portable photosynthesis system (Li-6400, Li-Cor, Lincoln, Nebraska, USA) with an air flow rate of 300 μmol s^−1^, external CO_2_ fixed at 400 μmol CO_2_ mol^−1^ and a red–blue light source attached to the leaf chamber with a PPFD of 1200 μmol m^−2^ s^−1^.

Chlorophyll fluorescence measurements were performed using a pulse-modulated field fluorescence monitoring system (FMS-2, Hansatech Instruments, Norfolk, UK). Leaves were previously adapted to darkness for 30 min and subsequently illuminated for 5 μs to calculate the ratio (F_m_ − F_0_)/F_m_. The chlorophyll fluorescence kinetics of leaves adapted to light were also studied. With all of the reaction centres closed, a pulse of saturating light (12,000 μmol m^−2^ s^−1^ for 0.8 s) was applied and, after that, the actinic light temporarily turned off and a pulse of far-red light (735 nm) was applied to drain the electrons from PSII.

### 4.5. Soil and Plant Mineral Analysis

Chemical characterisation of the substrate was performed at the end of the experiment [65]. The following parameters were analysed in a substrate/water (1/5) extract: EC, pH, exchangeable cations (Ca^2+^, Mg^2+^, K^+^, Na^+^), P, B, micronutrients (Fe, Mn, Cu, Zn), and anions (Cl^−^, SO_4_^2−^, NO_3_^−^, H_2_PO_4_^−^).

At the end of the experiment, a sample of fully expanded mature leaves was freeze-dried and ground for analytical determinations. Dried-plant tissues were ground, and an aliquot (250 mg) was ashed at 550 °C. Ashes were dissolved in 0.7 N HNO_3_, and phytotoxic elements (Na^+^ and B) were determined by coupled plasma optical emission spectrometry (Varian ICP-OES Vista MPX). Chloride was extracted from 50 mg of ground plant material with 2.5 mL of deionised water and measured by ion chromatography with a liquid chromatograph (Model ICS-3000, Thermo Fisher Scientific Inc., Logan, Utah, USA).

### 4.6. Osmolytes, Chlorophyll and H_2_O_2_ Determination

Proline was extracted from 50 mg of leaf tissue with sulfosalicylic acid (3%) and quantified according to the protocol described by Bates et al. [66]. Quaternary ammonium compounds (QAC) were extracted from dry tissue with 1 M H_2_SO_4_ and quantified using a glycine betaine standard curve, according to the method described in Grieve and Grattan [67].

Chlorophyll contents were estimated using the procedure described by Inskeep and Bloom [68], extracting 20 mg of ground material with N,N-dimethylformamide and measuring the absorbance at 664.5 and 647 nm in a Shimadzu UV-1800 spectrophotometer (Shimadzu Corporation, Kyoto, Japan).

Leaf H_2_O_2_ concentration was measured using the method of Velikova et al. [69] with minor modifications. Briefly, 0.25 g of fresh leaves were homogenised with 2.5 mL of 0.1% trichloroacetic acid in an ice bath and centrifuged at 12,000 × g for 15 min. Concentrations of H_2_O_2_ in the assay mixture (0.25 mL of supernatant, 0.25 mL of 10 mM potassium phosphate buffer pH 7.0, and 0.5 mL of 1M KI) were spectrophotometrically measured at 390 nm with an H_2_O_2_ standard curve.

### 4.7. Assay of Enzyme Activity Content

Enzyme extractions were collected following the method described by Noctor et al. [70]. Leaf tissues were homogenised in ice-cold in 0.1 M phosphate buffer, 1 mM EDTA (pH 7.5), followed by centrifugation at 12,000 rpm and 4 °C for 20 min. Supernatant was filtered with a 0.45 μm C_18_ Sep-Pak cartridge and was immediately used to determine the activities of the following enzymes:

Catalase (CAT). Its activity was determined spectrophotometrically by following the decrease in absorbance at 240 nm [70]. The mixture contained 840 μL of 0.1 M phosphate buffer, 1 mM EDTA (pH 7.5), and 100 μL of 400 mM H_2_O_2_. The reaction was initiated by adding 60 μL of enzyme extract.

Glutathione reductase (GR). The activity of GR was determined spectrophotometrically by following the decrease in absorbance at 340 nm [70]. The reaction mixture contained 880 μL of 0.1 M phosphate buffer, 1 mM EDTA (pH 7.5), 10 μL of 10 mM NADPH, and 100 μL of extract. The reaction was initiated by adding 10 μL of 50 mM GSSG.

Peroxidase. Peroxidase activity was determined spectrophotometrically by following the increase in absorbance at 470 nm [71]. The reaction mixture contained 700 μL of 0.1 M phosphate buffer, 1 mM EDTA (pH 7.5), 100 μL of 10 mM H_2_O_2_, 100 μL of 9 mM guaiacol, and 100 μL of enzyme extract.

Superoxide dismutase (SOD). Its activity was measured by the photochemical method as described by Fridovich [72]. One unit of SOD activity was defined as the amount of enzyme required to cause a 50% inhibition in the rate of nitro blue tetrazolium (NBT) reduction at 560 nm in the presence of riboflavin under the light. The reaction mixture contained 0.1 M phosphate buffer, 1 mM EDTA (pH 7.5), and 9.9 mM methionine, 57 μM NBT in ethanol, 0.9 μM riboflavin, 0.025% of triton and enzyme aliquot. Blanks were kept in the dark and the other samples were illuminated for 15 min.

Ascorbate peroxidase (APX). The extraction procedure was the same used for CAT, GR, peroxidase, and SOD, except for the extraction medium and the presence of 1 mM ascorbate [69]. APX activity was determined spectrophotometrically by following the decrease in absorbance at 290 nm. The reaction mixture contained 890 μL of 0.1 M phosphate buffer, 1 mM EDTA (pH 7.5), 50 μL of enzyme extract, 50 μL of 10 mM ascorbate, and 10 μL of 20 mM H_2_O_2_.

### 4.8. Endogenous Phytohormones

Samples of leaves were freeze-dried in liquid nitrogen and ground with a pestle into a coarse powder. Phytohormones were extracted from frozen leaves and analysed as previously described [73,74]. Powdered samples were homogenised with 80% methanol. Solids were separated by centrifugation and re-extracted with the same extraction solution. Pooled supernatants were passed through Sep-Pak C18 Plus short cartridges (SepPak Plus, Waters Corporation, Milford, MA, USA) to remove interfering lipids and part of the plant pigment, and, after removing the organic solvent by evaporation under vacuum, the residue was dissolved in 20% methanol and filtered through 13 mm diameter Millex filters with nylon membrane. The filtrated extracts were injected into a U-HPLC-MS system consisting of an Accela Series U-HPLC (ThermoFisher Scientific, Waltham, MA, USA) coupled to an Exactive mass spectrometer (ThermoFisher Scientific, Waltham, MA, USA) using a heated electrospray ionisation (HESI) interface. Mass spectra were obtained using the Xcalibur software, version 2.2 (ThermoFisher Scientific, Waltham, MA, USA). For the quantification of plant hormones, calibration curves were constructed for each analysed component and corrected for 10 μg L^−1^ deuterated internal standards. Hormone derivatives and conjugates were identified by extracting the exact mass from the full scan chromatogram obtained in negative mode and adjusting a mass tolerance of ≤1 ppm. Concentrations were semiquantitatively determined from the extracted peaks using calibration curves of analogue hormones.

### 4.9. Statistical Analysis

Data were analysed using analysis of variance (ANOVA) procedures in the Statgraphics Plus 5.1 software (Statistical Graphics Corporation, Warrenton, VA, USA). A two-way ANOVA procedure was used to discriminate the effects of the type of water and the irrigation method. When a significant effect was found (*p*-value < 0.05), means were separated using Duncan’s multiple range test.

## 5. Conclusions

Desalinated seawater is an alternative source of irrigation water in some areas with serious water scarcity and where situations of deficit irrigation may arise. Since citrus trees are widely grown in some of these areas (for example, Southeastern Spain), their cultivation under this scenario should be conducted with caution when DSW is used for irrigation. Citrus trees are sensitive to Cl^−^, Na^+^, and B, phytotoxic ions that are present in these waters, and their phytotoxicity problems could be aggravated when they are subject to periods of deficit irrigation since the high concentrations of phytotoxic elements in the soil and in the plant increase with DI (mainly Na^+^ and Cl^−^). The rootstock genotype that is used is of great importance since the citrus response to water stress and to the levels of Cl^−^, Na^+^, and B is rootstock dependent.

Both rootstocks irrigated with DSW and under DI showed different accumulations of phytotoxic elements, which influenced the plant behaviour: CM plants accumulated higher amounts of Na^+^ (above the threshold toxicity) and much more Cl^−^ (close to the threshold toxicity) than SO plants; however, the latter accumulated more B than CM (both above the threshold toxicity). According to that, Cl^−^, Na^+^, and B concentrations were high enough to produce foliar injuries in DSW-irrigated plants under DI, being Cl^−^ and Na^+^ in CM and B in SO the main causes of the decline in shoot growth of these plants.

Physiologically speaking, the behaviour of CM and SO plants was also different. Both rootstocks had a different response to the lowering of soil potential in DI and DSW treatments (lower water amount and higher salt concentration): CM plants maintained their cellular turgor due to a higher osmotic regulation (accumulation of Na^+^, Cl^−^, and proline), but SO failed to adjust osmotically to prevent turgor decrease (lower accumulation of Cl^−^ and proline than CM). In addition to the lower amount of chlorophyll in SO plants due to DSW and in CM plants due to DI, stomatal factors were implicated in the reduction of *A* in the first case, and alterations of the photochemical machinery participated in the second (the photochemical efficiency of PSII decreased due to damage in the light-harvesting complex). Under the most stressful conditions, both CM and SO plants increased their NPQ in order to protect the PSII reaction centres from harmful excess excitation energy. Finally, unlike CM plants, SO plants irrigated with DSW and under DI had a good antioxidant system and did not generate MDA, since they were able to cope with the ROS produced by the excess of electrons that was generated.

The obtained data verified that each rootstock had a different response to the stress generated by the irrigation with DSW and/or by DI, and this response was dependent on the phytotoxic elements accumulated in the leaves, as well as its specific toxicity. Due to the changing environment that has emerged as a result of climate change, situations of water stress could occur in crops irrigated with DSW. In this case, knowing the behaviour of different genetic materials could be relevant to decide which plant material to use when temperatures rise due to CC and farmers are forced to use DSW for citrus irrigation under water shortage situations.

## Figures and Tables

**Table 1:** Soil chemical properties for the initial soil (before the experiment) and at the end of the experiment, after seven months of irrigation with water of different characteristics: Control and DSW and after 75 days of application of two irrigation regimens (full irrigation (FI) or deficit irrigation (DI)).of lemon citrus plants under *Citrus macrophylla* or sour orange rootstocks, irrigated with Control or DSW and under two EC in μS cm^−1^; and nutrients in mg kg^−1^.

		EC	Na^+^	Cl^−^	B	K^+^	Mg^2+^	Ca^2+^	SO_4_^2−^	NO_3_^−^
		BEFORE THE EXPERIMENT								
		206 ± 16	59.8 ± 12	61 ± 14	0.30 ± 0.01	23 ± 1	16.5 ± 0.6	115 ± 3	57 ± 6	17 ± 2
Type of water (TW)		END OF THE EXPERIMENT								
		*Citrus macrophylla* (CM)								
Control		376 ± 51	33.4 ± 7.8	34 ± 15	0.33 ± 0.02	179 ± 29	26.3 ± 3.8	167 ± 23	137 ± 22	501 ± 140
DSW		491 ± 64	144.1 ± 24.9	193 ± 52	1.26 ± 0.02	180 ± 19	26.8 ± 3.7	168 ± 20	153 ± 19	522 ± 102
Irrigation (I)										
FI		303 ± 22	57.7 ± 12	74 ± 15	0.70 ± 0.15	131 ± 8	18.1 ± 1.6	120 ± 7	106 ± 9	242 ± 36
DI		564 ± 49	119.8 ± 33.7	153 ± 52	0.90 ± 0.22	228 ± 18	35.0 ± 2.3	215 ± 15	184 ± 18	781 ± 89
TW × I										
Control	FI	258 ± 22	31.2 ± 11.1 a	42 ± 8	0.30 ± 0.03	120 ± 6	18.1 ± 3.0	116 ± 12	98 ± 19	169 ± 17
	DI	493 ± 52	35.7 ± 12.6 a	26 ± 9	0.36 ± 0.03	239 ± 30	34.5 ± 3.5	217 ± 25	176 ± 31	834 ± 131
DSW	FI	347 ± 20	84.3 ± 9.2 b	106 ± 11	1.09 ± 0.08	141 ± 13	18.1 ± 1.8	123 ± 7	114 ± 4	316 ± 47
	DI	634 ± 73	203.9 ± 20.7 c	280 ± 40	1.44 ± 0.16	218 ± 25	35.4 ± 3.4	213 ± 22	192 ± 24	729 ± 134
ANOVA										
TW		*	***	***	***	ns	ns	ns	ns	ns
I		***	***	**	*	**	***	***	**	***
TW × I		ns	**	**	ns	ns	ns	ns	ns	ns
Type of water (TW)		Sour orange (SO)								
Control		447 ± 62	41.7 ± 8.6	35 ± 10	0.32 ± 0.02	199 ± 25	28.4 ± 4.4	192 ± 27	167 ± 28	609 ± 132
DSW		472 ± 61	127.6 ± 17.0	192 ± 29	0.90 ± 0.07	182 ± 17	24.6 ± 3.0	173 ± 18	145 ± 19	483 ± 98
Irrigation (I)										
FI		318 ± 24	68.1 ± 11.5	89 ± 21	0.53 ± 0.10	143 ± 14	18.4 ± 1.7	137 ± 11	118 ± 15	261 ± 44
DI		600 ± 32	101.2 ± 26.1	138 ± 46	0.69 ± 0.13	238 ± 10	34.7 ± 2.9	228 ± 19	194 ± 23	830 ± 55
TW × I										
Control	FI	305 ± 43	48.1 ± 14.8 a	46 ± 17 a	0.28 ± 0.02	140 ± 23	18.4 ± 3.3	134 ± 20	117 ± 27	279 ± 80
	DI	589 ± 19	35.4 ± 9.9 a	24 ± 7 a	0.36 ± 0.04	258 ± 8	38.4 ± 3.8	250 ± 27	217 ± 36	939 ± 43
DSW	FI	332 ± 27	88.1 ± 11.5 b	132 ± 25 b	0.79 ± 0.08	146 ± 19	18.3 ± 1.4	139 ± 12	119 ± 20	244 ± 47
	DI	611 ± 58	167.1 ± 13.6 c	252 ± 31 c	1.02 ± 0.07	219 ± 12	30.9 ± 3.7	206 ± 25	171 ± 28	722 ± 66
ANOVA										
TW		ns	***	***	***	ns	ns	ns	ns	ns
I		***	*	*	*	**	***	***	*	***
TW × I		ns	**	**	ns	ns	ns	ns	ns	ns

* *p* < 0.05; ** *p* < 0.01; *** *p* < 0.001; ns: not significant. Different letters indicate significant differences according to Duncan’s multiple range test at the 95% confidence level.

**Table 2:** Average ionic composition and electrical conductivity (EC) of the nutrient solutions in the Control and DSW (desalinated seawater) treatments used for irrigation during the experiment.

	Control	DSW
EC (μS cm^−1^)	2000	3079
Cl^−^ (mg L^−1^)	1.8	300.1
Na^+^ (mg L^−1^)	0.0	166.6
B (mg L^−1^)	0.27	1.23
NO_3_^−^ (mg L^−1^)	992	1003
H_2_PO_4_^−^ (mg L^−1^)	192	192
SO_4_^2−^ (mg L^−1^)	96.0	108.3
K^+^ (mg L^−1^)	235.0	240.3
Ca^2+^ (mg L^−1^)	160.0	179.3
Mg^2+^ (mg L^−1^)	24.0	28.6
Cu (mg L^−1^)	0.032	0.044
Zn (mg L^−1^)	0.131	0.131
Mn (mg L^−1^)	0.11	0.11
Fe (mg L^−1^)	1.12	1.12

**Table 3:** Plant growth of “Verna” lemon plants grafted on *Citrus macrophylla* (CM) or sour orange (SO) rootstocks, irrigated with two types of water (Control or desalinated seawater (DSW)) and under two irrigation regimens (full irrigation (FI) or deficit irrigation (DI)).

		Dry Weight (g)					Root/ Shoot	New Stem Length	Foliar Area (cm^2^)	Mean Leaf Area (cm^2^)	Number of Leaves	Damaged Leaves (%)
Type of Water (TW)		Leaves	New Stem	Shoot	Root	Plant						
		*Citrus macrophylla* (CM)										
Control		36.1	14.8	69.2	17.0	86.2	0.26	176	3987	31.7	125	0.9
DSW		30.0	10.3	59.2	18.2	76.8	0.31	185	3353	26.5	126	15.3
Irrigation (I)												
FI		38.7	13.7	71.6	15.4	87.0	0.22	198	4496	31.3	144	8.1
DI		27.4	11.4	56.9	19.7	76.0	0.35	164	2844	26.9	106	8.1
TW × I												
Control	FI	42.8	16.6	79.3	15.0	94.3	0.19	195	4951	34.3	146	0.3
	DI	29.5	13.0	59.2	19.0	78.2	0.32	158	3023	29.1	104	1.5
DSW	FI	34.5	10.8	63.9	15.9	79.7	0.25	200	4041	30.7	143	15.8
	DI	25.4	9.9	54.6	20.4	75.0	0.38	169	2665	24.7	109	14.7
ANOVA												
TW		*	**	*	ns	ns	ns	ns	*	*	ns	***
I		***	ns	**	ns	ns	**	*	***	ns	***	ns
TW × I		ns	ns	ns	ns	ns	ns	ns	ns	ns	ns	ns
Type of water (TW)		Sour orange (SO)										
Control		32.1	15.4	72.7	22.3	95.0	0.31	178	3196	29.1	112	0.9
DSW		28.6	11.6	56.7	19.8	74.7	0.32	140	2894	29.8	89	18.8
Irrigation (I)												
FI		35.1	15.1	68.2	20.8	87.3	0.28	171	3585	29.1	115	9.7
DI		25.7	11.9	61.1	21.3	82.4	0.35	148	2505	29.8	86	10.0
TW × I												
Control	FI	35.6	16.3	77.0	20.0	97.0	0.26	203	3649	30.0	126	0.3
	DI	28.6	14.4	68.4	24.5	92.9	0.36	154	2743	28.2	98	1.5
DSW	FI	34.5	13.9	59.4	21.5	77.5	0.31	138	3521	28.3	105	19.1
	DI	22.8	9.3	53.9	18.0	71.9	0.34	143	2267	31.4	74	18.5
ANOVA												
TW		ns	*	*	ns	ns	ns	ns	ns	ns	ns	***
I		**	*	*	ns	ns	*	ns	**	ns	*	ns
TW × I		ns	ns	ns	ns	ns	ns	ns	ns	ns	ns	ns

* *p* < 0.05; ** *p* < 0.01; *** *p* < 0.001; ns: not significant.

**Table 4:** Photosynthesis rate (*A*), stomatal conductance (*g_s_*), transpiration (*E*), efficiency of the antennas from PSII (F’_v_/F’_m_), photochemical efficiency of PSII (Φ_PSII_), photochemical quenching (qP), non-photochemical quenching (NPQ), A/Φ_PSII_ ratio and total chlorophyll in leaves of lemon citrus plants under *Citrus macrophylla* or sour orange rootstocks, irrigated with Control or DSW and under two irrigation regimens (full irrigation (FI) or deficit irrigation (DI)) at the end of the experiment.

Type of Water (TW)		*A* (μmol m^−2^ s^−1^)	*g_s_* (mol m^−2^ s^−1^)	*E* (mmol m^−2^ s^−1^)	F^’^_v_/F^’^_m_	Φ_PSII_	qP	NPQ	A/Φ_PSII_	Chlorophyll (mg g^−1^ DW)
		*Citrus macrophylla* (CM)								
Control		2.12	0.0092	0.472	0.634	0.487	0.763	1.925	4.23	9.8
DSW		1.82	0.0076	0.282	0.665	0.541	0.804	1.077	3.40	10.1
Irrigation (I)										
FI		2.25	0.0091	0.418	0.699	0.566	0.809	1.489	3.72	10.7
DI		1.69	0.0076	0.336	0.600	0.462	0.758	1.513	3.91	9.2
TW × I										
Control	FI	2.57	0.0087	0.506	0.698	0.541	0.773	2.292 c	4.22	10.3
	DI	1.66	0.0096	0.438	0.570	0.433	0.752	1.558 b	4.25	9.2
DSW	FI	1.92	0.0096	0.331	0.700	0.591	0.844	0.685 a	3.22	11.0
	DI	1.73	0.0056	0.234	0.630	0.492	0.764	1.468 b	3.57	9.2
ANOVA										
TW		ns	ns	ns	ns	ns	ns	***	*	ns
I		*	ns	ns	**	*	ns	ns	ns	**
TW × I		ns	ns	ns	ns	ns	ns	***	ns	ns
Type of water (TW)		Sour orange (SO)								
Control		2.05	0.0085	0.346	0.651	0.517	0.786	1.017	4.11	9.4
DSW		1.36	0.0052	0.130	0.698	0.584	0.810	1.353	2.38	8.2
Irrigation (I)										
FI		1.68	0.0070	0.250	0.765	0.630	0.808	1.065	2.89	9.2
DI		1.72	0.0068	0.226	0.583	0.471	0.788	1.304	3.59	8.5
TW × I										
Control	FI	2.15	0.0085	0.340	0.756	0.606	0.774	0.860	3.92	9.8
	DI	1.94	0.0085	0.352	0.545	0.428	0.798	1.174	4.30	9.1
DSW	FI	1.21	0.0054	0.160	0.774	0.654	0.843	1.271	1.87	8.5
	DI	1.51	0.0051	0.100	0.622	0.514	0.777	1.435	2.88	7.8
ANOVA										
TW		*	ns	*	ns	ns	ns	*	*	*
I		ns	ns	ns	*	ns	ns	ns	ns	ns
TW × I		ns	ns	ns	ns	ns	ns	ns	ns	ns

* *p* < 0.05; ** *p* < 0.01; *** *p* < 0.001; ns: not significant. Different letters indicate significant differences according to Duncan’s multiple range test at the 95% confidence level.

**Table 5:** Concentration of ascorbate peroxidase (APX), catalase (CAT), peroxidase, glutathione reductase (GR), and superoxide dismutase (SOD) on leaves of lemon citrus plants under *Citrus macrophylla* or sour orange rootstocks, irrigated with Control or DSW and under two irrigation regimens (full irrigation (FI) or deficit irrigation (DI)) at the end of the experiment.

Type of Water (TW)		APX (nmol mg^−1^ Protein min^−1^)	CAT (μmol mg^−1^ Protein min^−1^)	Peroxidase (nmol mg^−1^ Protein min^−1^)	GR (nmol mg^−1^ Protein min^−1^)	SOD (U mg^−1^ Protein)
		*Citrus macrophylla* (CM)				
Control		3017	74.3	912	146.5	2217
DSW		3075	64.6	846	144.5	2024
Irrigation (I)						
FI		3052	74.2	964	128.9	2326
DI		3040	67.8	795	162.1	1915
TW × I						
Control	FI	3064	78.0	1032	134.2	2841
	DI	2970	70.5	791	158.7	1811
DSW	FI	3041	70.4	895	123.6	1593
	DI	3109	58.8	798	165.5	2236
ANOVA						
TW		ns	ns	ns	ns	ns
I		ns	ns	ns	ns	ns
TW × I		ns	ns	ns	ns	ns
Type of water (TW)		Sour orange (SO)				
Control		2695	52.7	715	109.8	2021
DSW		3580	62.9	878	135.1	1573
Irrigation (I)						
FI		2746	66.9	914	137.1	1601
DI		3529	48.7	679	107.8	1992
TW × I						
Control	FI	2379	61.2	833	124.4	1791
	DI	3010	44.2	598	95.3	1411
DSW	FI	3113	72.6	994	149.8	2251
	DI	4047	53.2	761	120.3	1734
ANOVA						
TW		*	ns	*	ns	*
I		*	*	*	ns	*
TW × I		ns	ns	ns	ns	ns

* *p* < 0.05; ns: not significant.

**Table 6:** Endogenous levels of 1-aminocyclopropane 1-carboxylic acid (ACC), trans-zeatin (tZ), gibberellic acid 3 (GA3), gibberellin A4 (GA4), indole-3-acetic acid (IAA), abscisic acid (ABA), jasmonic acid (JA), and salicylic acid (SA) measured at the end of the experiment in leaves of lemon citrus plants under *Citrus macrophylla* or sour orange rootstocks, irrigated with Control or DSW and under two irrigation regimens (full irrigation (FI) or deficit irrigation (DI)).

Type of Water (TW)		ACC (ng g^−1^ FW)	tZ (ng g^−1^ FW)	GA3 (ng g^−1^ FW)	GA4 (ng g^−1^ FW)	IAA (ng g^−1^ FW)	ABA (ng g^−1^ FW)	JA (ng g^−1^ FW)	SA (ng g^−1^ FW)
		*Citrus macrophylla* (CM)							
Control		276.8	188.2	0.410	0.016	0.154	7.70	8.78	10.16
DSW		358.4	136.9	0.643	0.011	0.221	6.93	9.09	10.31
Irrigation (I)									
FI		332.0	161.8	0.451	0.025	0.208	7.46	7.24	11.12
DI		303.2	163.3	0.601	0.003	0.166	7.17	10.63	9.35
TW × I									
Control	FI	305.7	185.9	0.363	0.027	0.157	7.65	5.86	10.85
	DI	247.8	190.5	0.456	0.005	0.150	7.74	11.70	9.47
DSW	FI	358.2	137.8	0.539	0.022	0.259	7.26	8.62	11.39
	DI	358.5	136.1	0.746	0.000	0.182	6.60	9.56	9.23
ANOVA									
TW		ns	**	ns	ns	**	ns	ns	ns
I		ns	ns	ns	**	ns	ns	ns	ns
TW × I		ns	ns	ns	ns	ns	ns	ns	ns
Type of water (TW)		Sour orange (SO)							
Control		318.0	192.7	0.494	0.018	0.197	7.47	9.77	12.92
DSW		292.8	195.9	0.601	0.009	0.243	6.57	7.62	11.40
Irrigation (I)									
FI		324.4	200.4	0.435	0.007	0.204	7.77	11.27	10.87
DI		286.4	188.2	0.660	0.020	0.236	6.27	6.13	13.45
TW × I									
Control	FI	343.7	247.5 b	0.407	0.012	0.209	8.86	12.51	12.87
	DI	292.3	137.9 a	0.581	0.024	0.185	6.08	7.04	12.97
DSW	FI	305.1	153.3 a	0.464	0.003	0.199	6.68	10.03	8.87
	DI	280.5	238.5 ab	0.738	0.015	0.288	6.46	5.22	13.92
ANOVA									
TW		ns	ns	ns	ns	ns	ns	ns	ns
I		ns	ns	ns	ns	ns	ns	ns	ns
TW × I		ns	*	ns	ns	ns	ns	ns	ns

* *p* < 0.05; ** *p* < 0.01; ns: not significant. Different letters indicate significant differences according to Duncan’s multiple range test at the 95% confidence level.

## Data Availability

Data will be made available upon request.
